# Supplementary material for: Inhibiting Leishmania donovani Sterol Methyltransferase to Identify Lead Compounds Using Molecular Modelling
Source: Pharmaceuticals (Basel). 2023 Feb 21;16(3):330. doi: 10.3390/ph16030330 (PMC10054574; doi:10.3390/ph16030330)
Supplement: Supplementary file 1 [file pharmaceuticals-16-00330-s001.zip › pharmaceuticals-2009824-supplementary.pdf]

SUPPLEMENTARY DATA

(a)

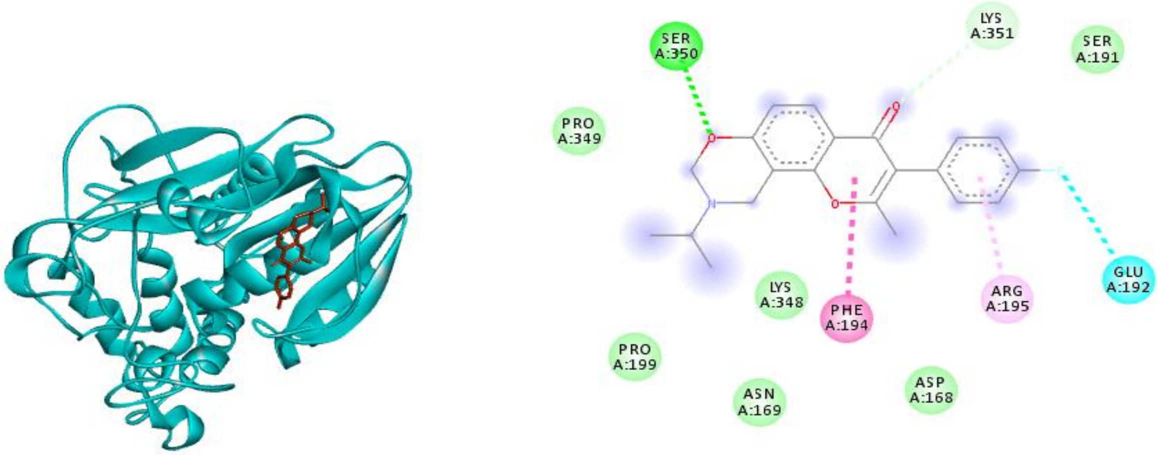

(b)

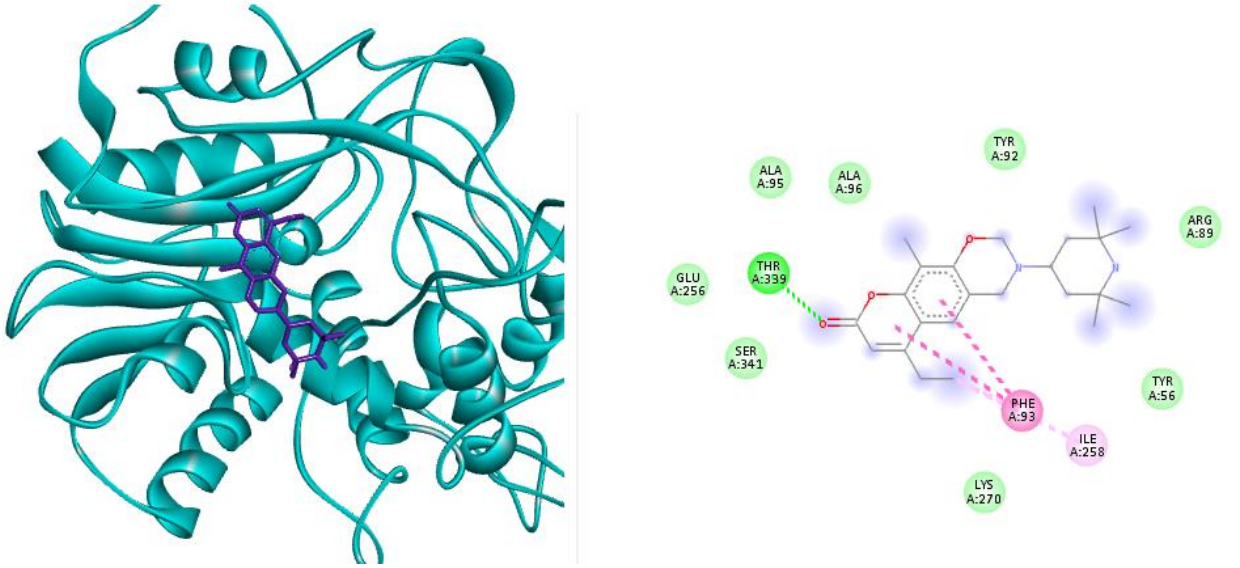

(C)

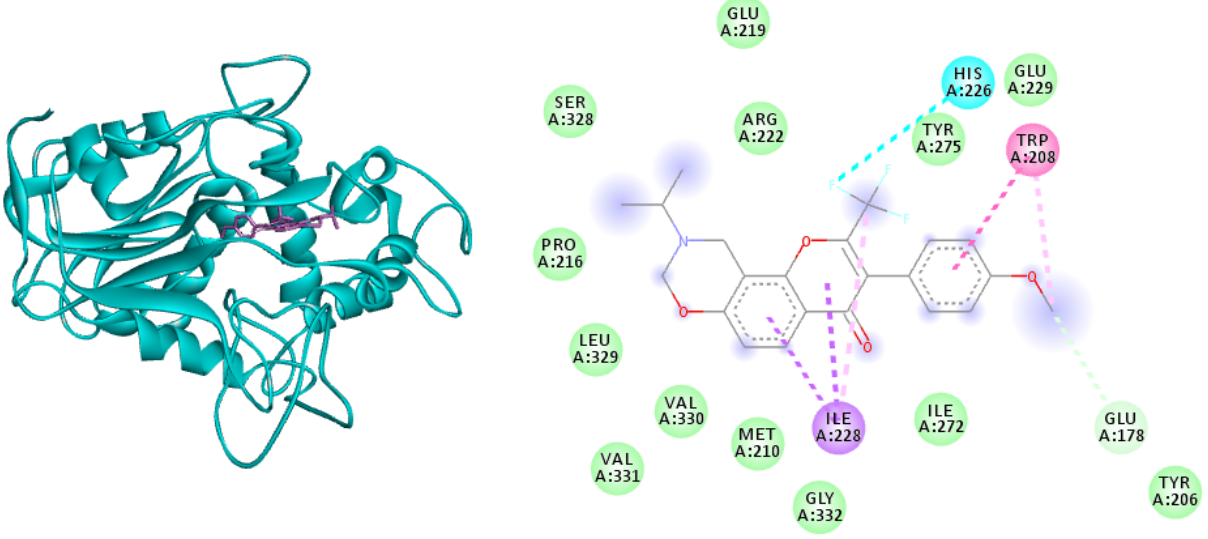
GLU A:219
SER A:328
ARG A:222
HIS A:226
GLU A:229
TYR A:275
TRP A:208
PRO A:216
LEU A:329
VAL A:330
MET A:210
ILE A:228
ILE A:272
GLU A:178
VAL A:331
GLY A:332
TYR A:206

(d)

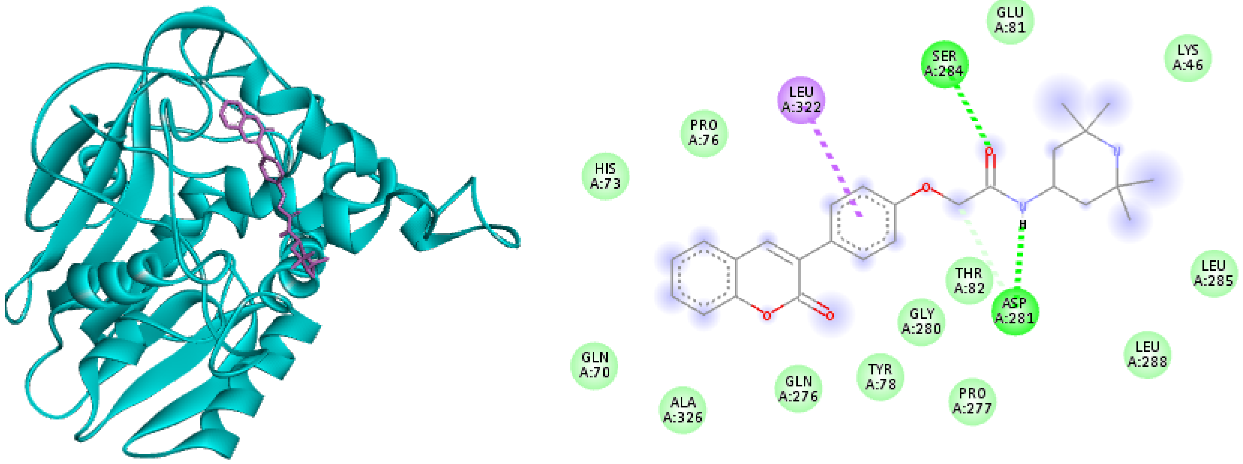
GLU A:81
LYS A:46
SER A:284
LEU A:322
PRO A:76
HIS A:73
THR A:82
ASP A:281
GLY A:280
LEU A:285
LEU A:288
GLN A:70
ALA A:326
GLN A:276
TYR A:78
PRO A:277

(e)

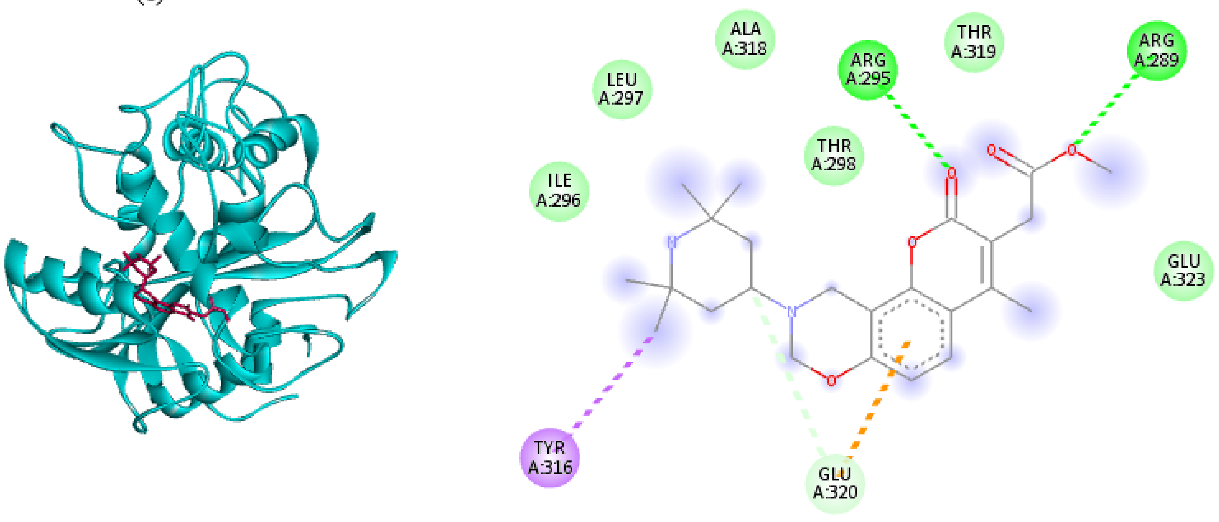
ALA A:318
LEU A:297
ARG A:295
THR A:319
ARG A:289
ILE A:296
THR A:298
GLU A:323
TYR A:316
GLU A:320

(f)

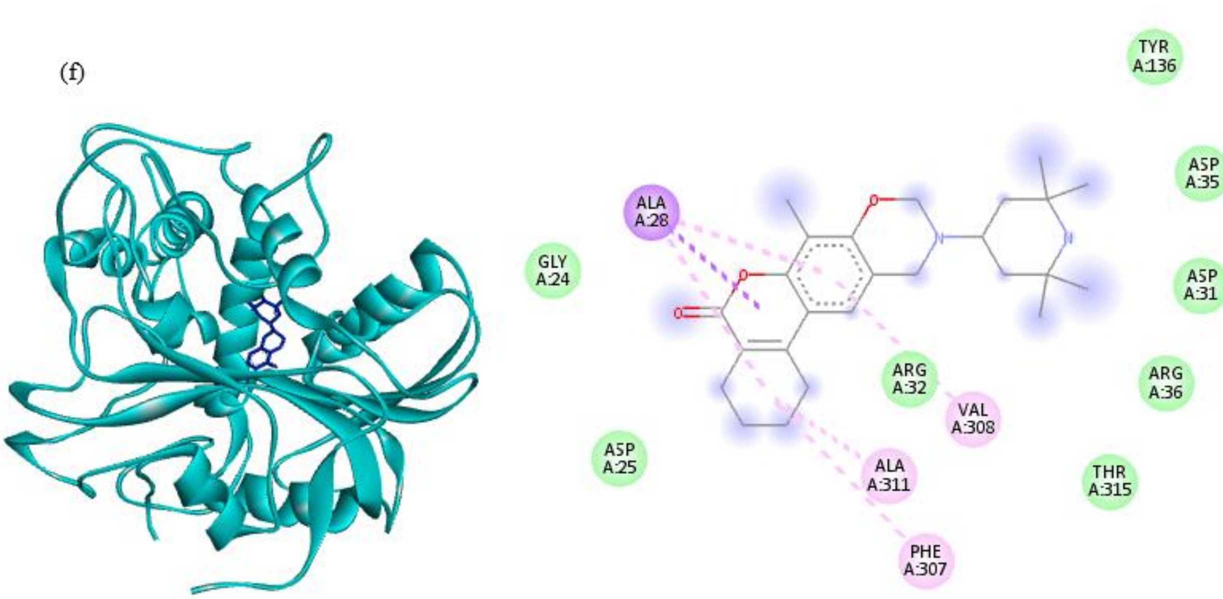
TYR A:136
ASP A:35
ALA A:28
GLY A:24
ASP A:31
ARG A:32
ARG A:36
VAL A:308
ASP A:25
ALA A:311
THR A:315
PHE A:307

(g)

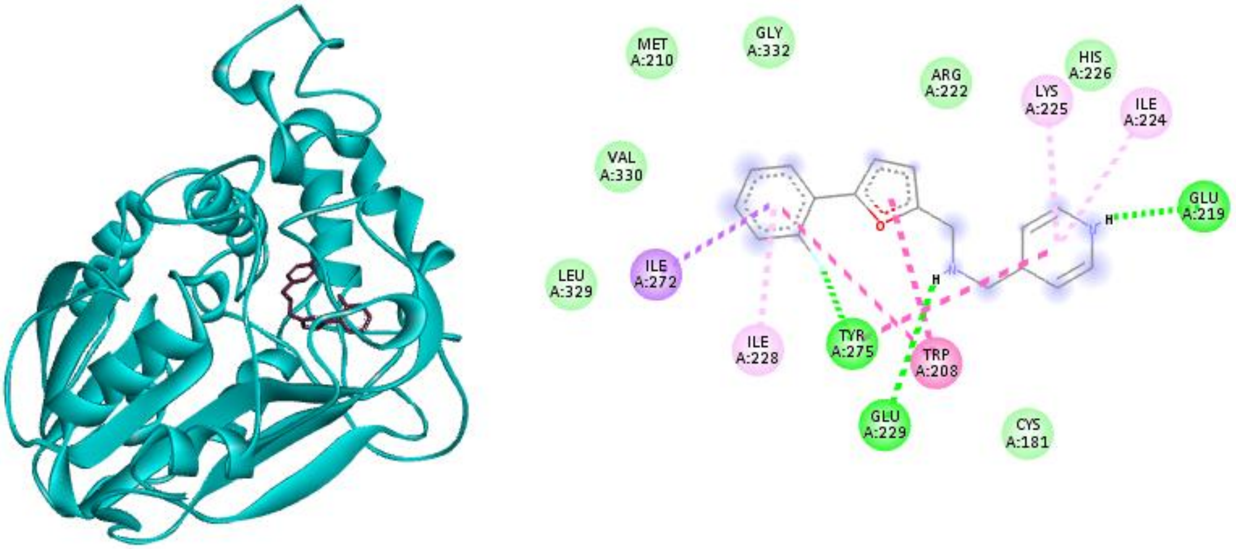
MET
A:210
GLY
A:332
ARG
A:222
HIS
A:226
LYS
A:225
ILE
A:224
VAL
A:330
GLU
A:219
LEU
A:329
ILE
A:272
ILE
A:228
TYR
A:275
TRP
A:208
GLU
A:229
CYS
A:181

(h)

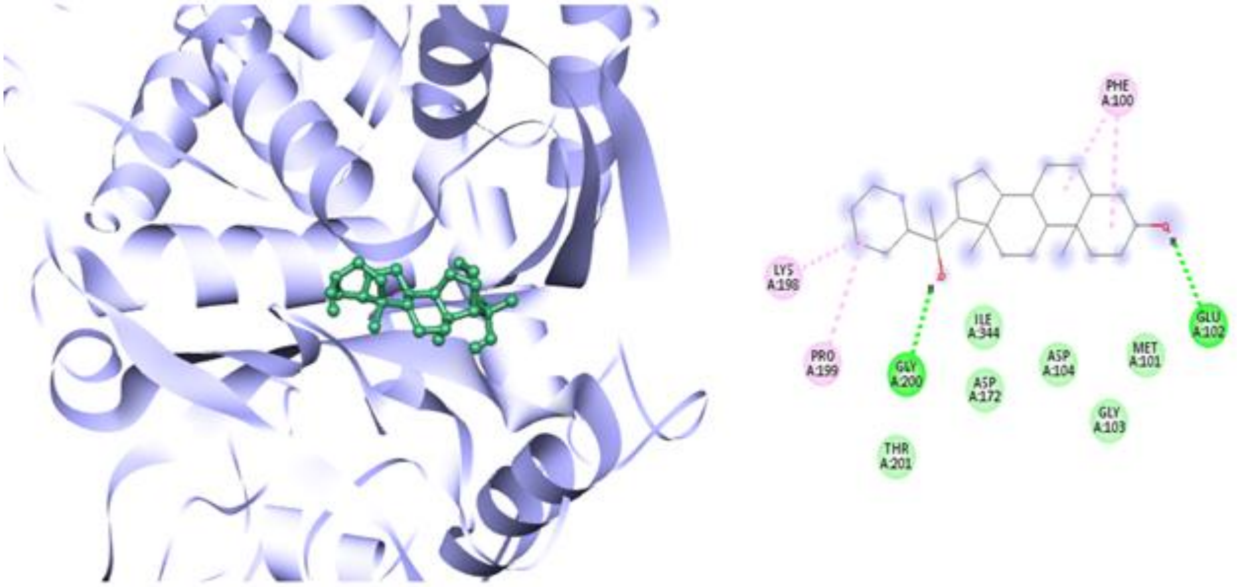
PHE
A:100
LYS
A:198
PRO
A:199
GLY
A:200
ILE
A:344
ASP
A:172
ASP
A:104
MET
A:101
GLY
A:103
GLU
A:102
THR
A:201

(i)

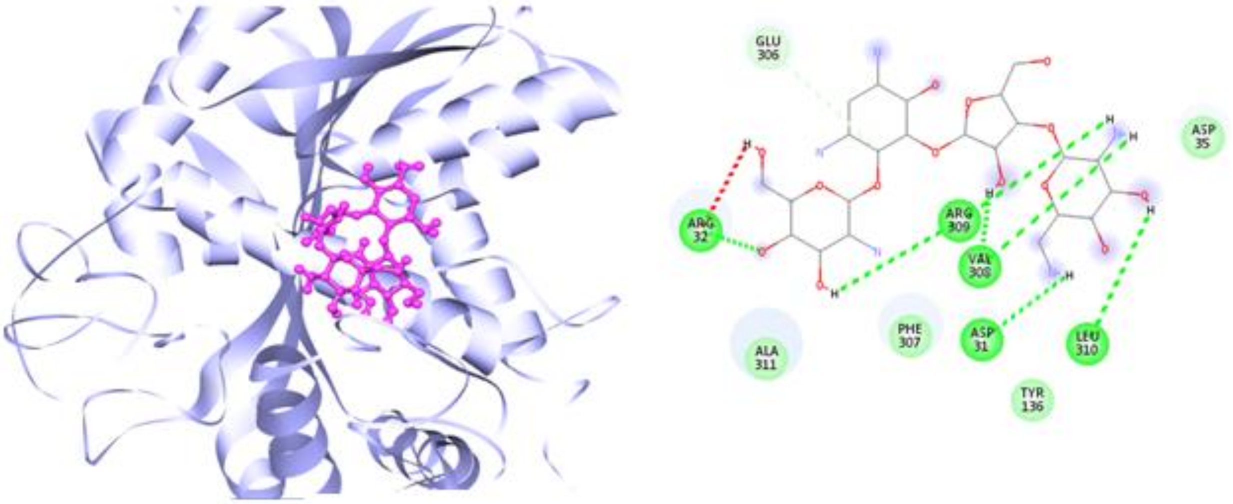
GLU 306
ASP 35
ARG 32
ARG 309
VAL 308
ALA 311
PHE 307
ASP 31
LEU 310
TYR 136

(j)

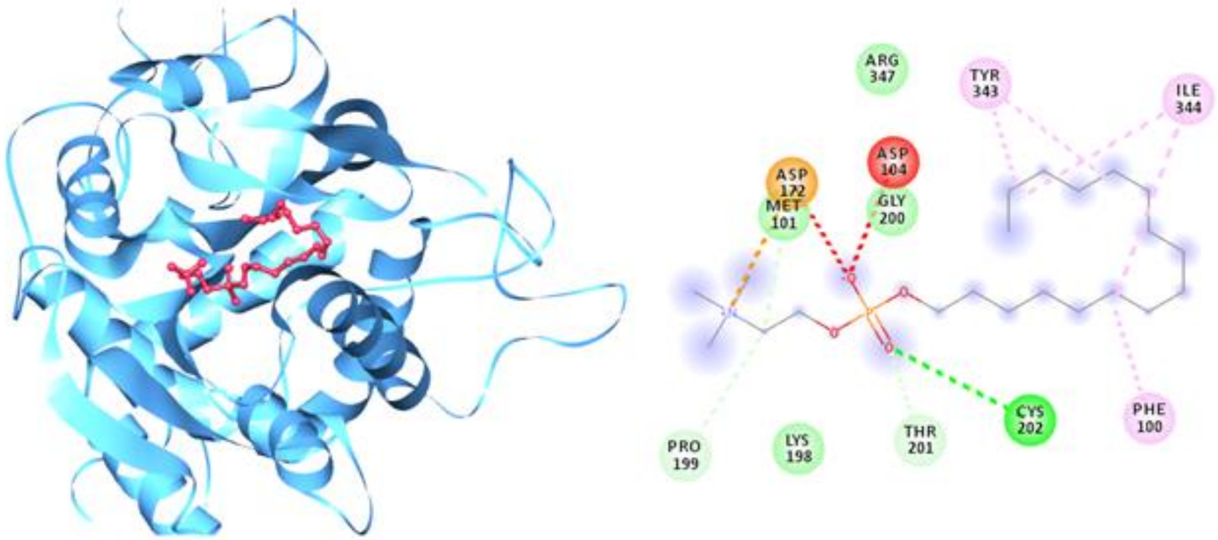
ARG 347
TYR 343
ILE 344
ASP 172
ASP 104
MET 101
GLY 200
PRO 199
LYS 198
THR 201
CYS 202
PHE 100

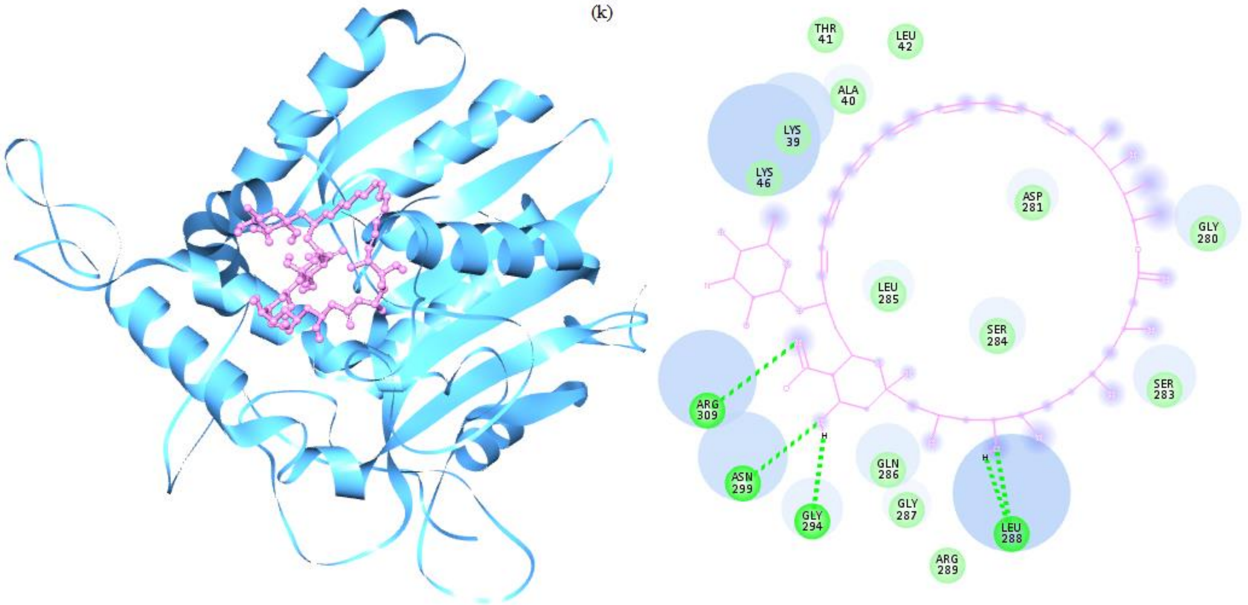

Figure S1. The cartoon representations and 2D interactions of the *Ld*SMT-hits complexes as visualized in Discovery Studio v19.1.0.18287. It shows complexes of (a) *Ld*SMT-STOCK6S-65920, (b) *Ld*SMT-STOCK6S-16994, (c) *Ld*SMT-STOCK6S-55084, (d) *Ld*SMT-STOCK6S-64941, (e) *Ld*SMT-STOCK6S-19430, (f) *Ld*SMT-STOCK6S-14893, (g) *Ld*SMT-STOCK6S-07353, (h) *Ld*SMT-22,26-Azasterol, (i) *Ld*SMT-paromomycin, (j) *Ld*SMT-miltefosine, and (k) *Ld*SMT-amphotericin B.

Table S1. Biological activity predictions of selected hit compounds with their probability of activity (Pa) and probability of inactivity (Pi).

Selected Hits	Antineoplastic activities		Alzheimer treatment		Dermatologic		Aspulvinone dimethylallyltransferase inhibitor		Indolepyruvate C-methyltransferase inhibitor		Lanosterol 14alpha demethylase inhibitor		Cyclin-dependent kinase 12 inhibitor	
	Pa	Pi	Pa	Pi	Pa	Pi	Pa	Pi	Pa	Pi	Pa	Pi	Pa	Pi
STOCK6S-06707	0.368	0.117	0.565	0.008	0.236	0.130	-	-	-	-	-	-	-	-
STOCK6S-84928	0.566	0.005	-	-	0.196	0.170	-	-	-	-	-	-	-	-
STOCK6S-65920	0.312	0.145	-	-	-	-	-	-	-	-	0.147	0.032	0.47	0.032
STOCK6S-55084	0.542	0.059	0.243	0.102	-	-	-	-	-	-	0.143	0.037	0.253	0.215
STOCK6S-64941	0.239	0.194	-	-	-	-	0.435	0.170	-	-	-	-	-	-
STOCK6S-19430	0.258	0.180	0.359	0.041	-	-	-	-	-	-	-	-	-	-
STOCK6S-14893	0.429	0.094	0.598	0.006	0.283	0.097	-	-	-	-	-	-	-	-
STOCK6S-07353	0.209	0.021	-	-	-	-	-	-	0.156	0.142	-	-	-	-
STOCK6S-16994	0.339	0.131	0.513	0.011	0.351	0.066	-	-	-	-	-	-	-	-

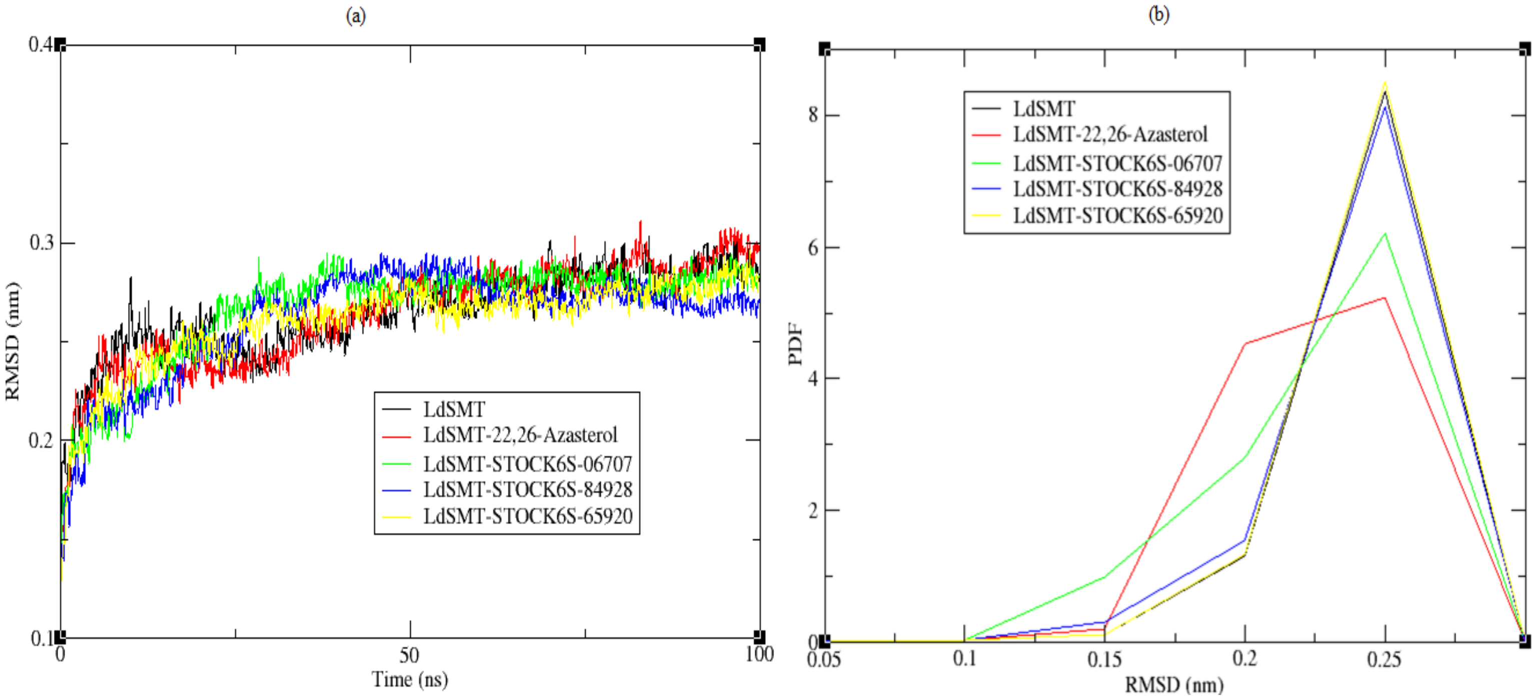

Figure S2. The RMSD and PDF analysis of 100 ns trajectory of the unbound *Ld*SMT, complexes of the three selected hits and 22,26-Azasterol.. (a) RMSD for unbound protein and all the complexes, (b) PDF of RMSD of unbound *Ld*SMT, *Ld*SMT-22,26-Azasterol, *Ld*SMT-STOCK6S-06707, *Ld*SMT-STOCK6S-84928 and *Ld*SMT-STOCK6S-65920.

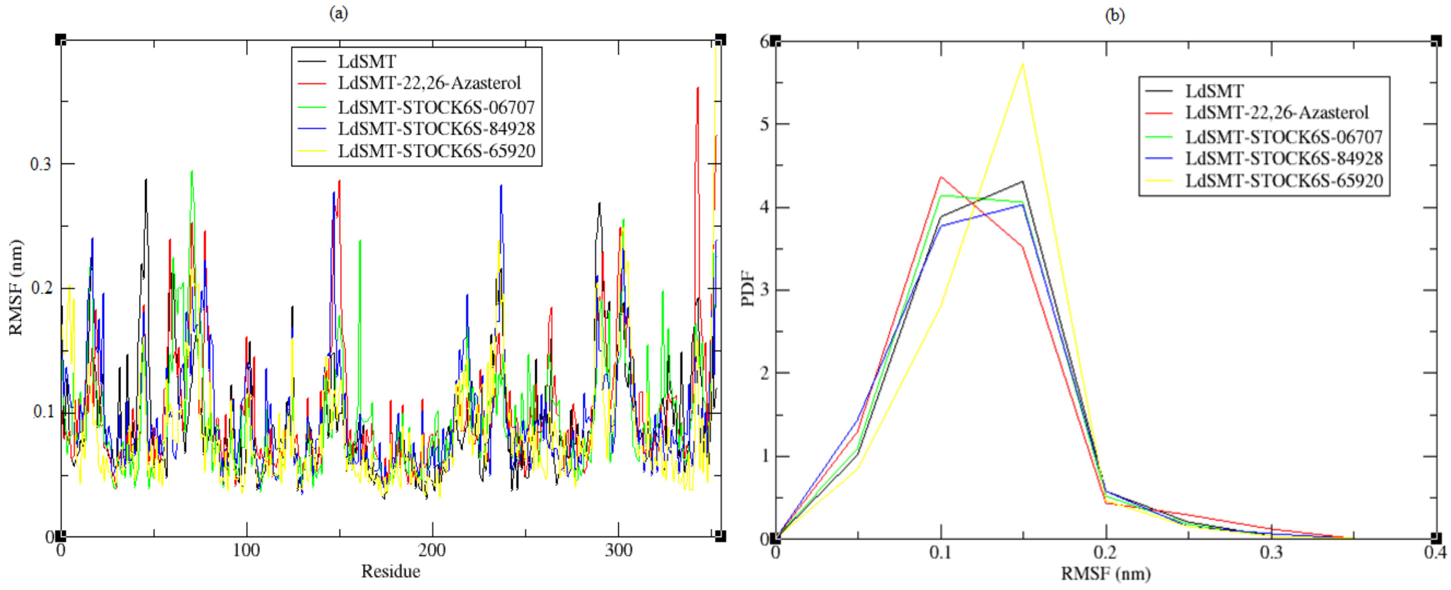

Figure S3. (a) Combined RMSF plot for the unbound *Ld*SMT and the protein-ligand complexes and (b) PDF of RMSF unbound *Ld*SMT, *Ld*SMT-22,26-Azasterol, *Ld*SMT-STOCK6S-06707, *Ld*SMT-STOCK6S-84928 and *Ld*SMT-STOCK6S-65920)

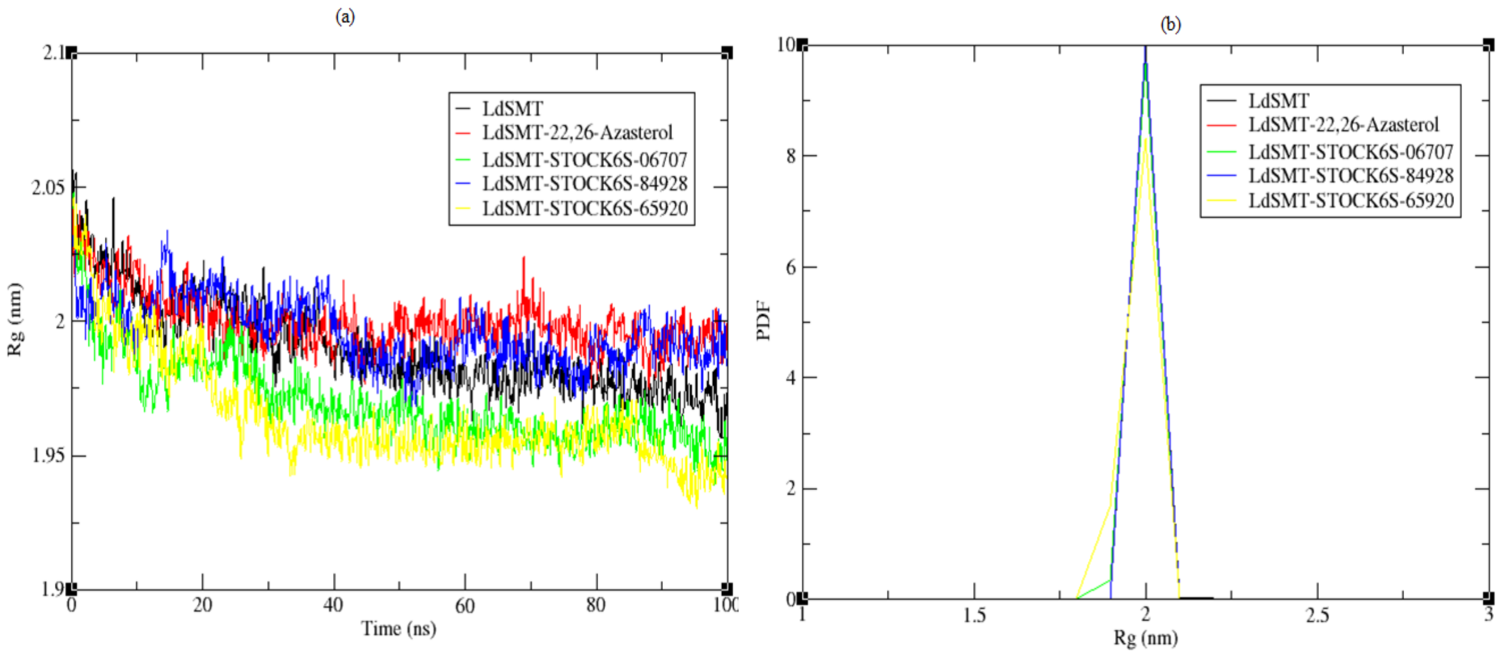

Figure S4. Rg analysis of 100 ns trajectory of the unbound *Ld*SMT, complexes of the three selected hits and 22,26-Azasterol. (a) Rg for unbound protein and all the complexes, (b) PDF of Rg of *Ld*SMT, *Ld*SMT-22,26-Azasterol, *Ld*SMT-STOCK6S-06707, *Ld*SMT-STOCK6S-84928, and *Ld*SMT-STOCK6S-65920).

(a)

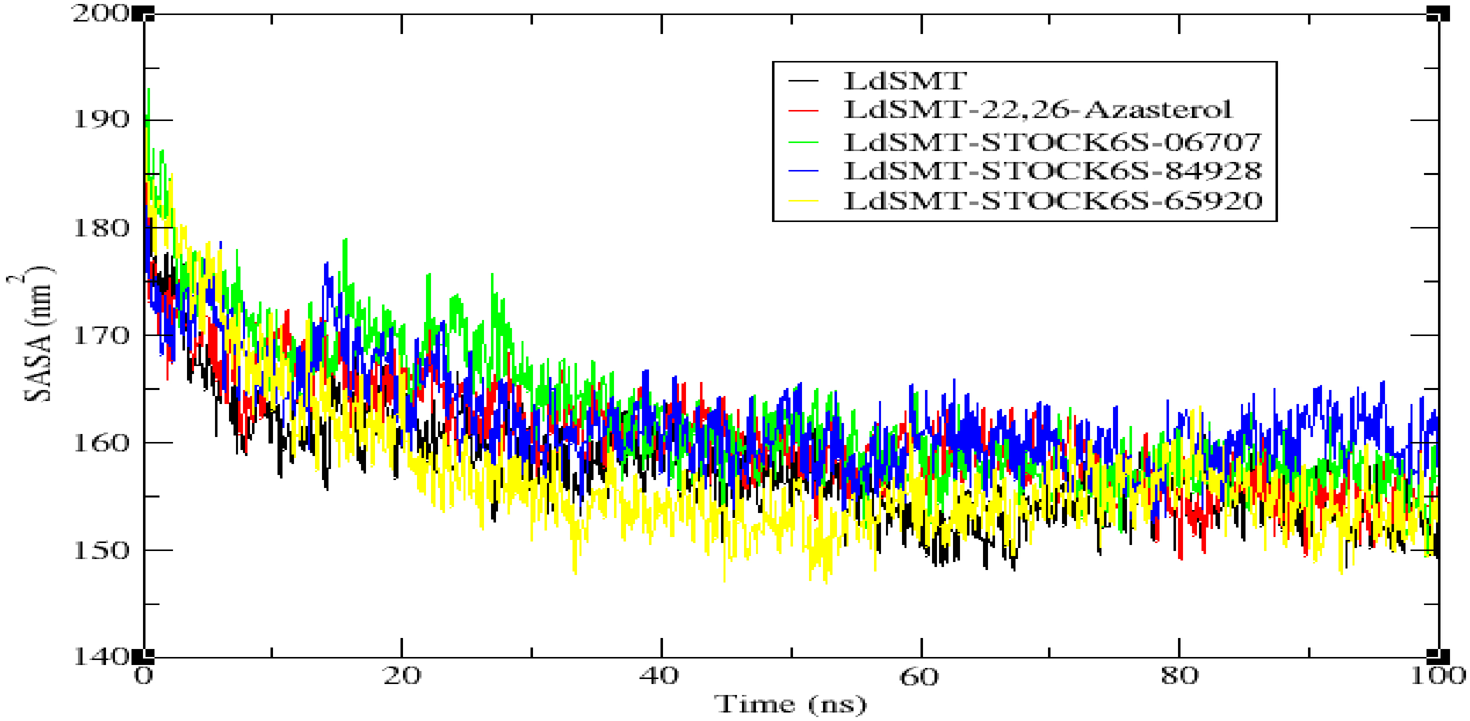

LdSMT
LdSMT-22,26-Azasterol
LdSMT-STOCK6S-06707
LdSMT-STOCK6S-84928
LdSMT-STOCK6S-65920
SASA (nm²)
Time (ns)

(b)

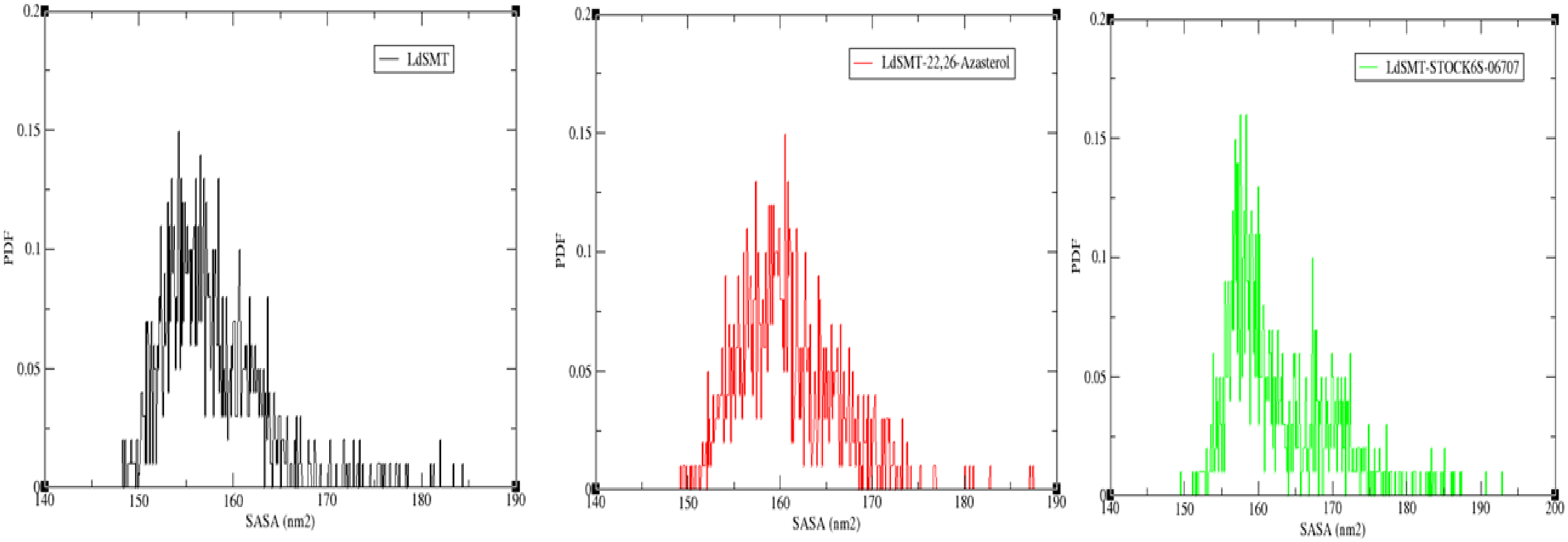

LdSMT
LdSMT-22,26-Azasterol
LdSMT-STOCK6S-06707
PDF
SASA (nm2)

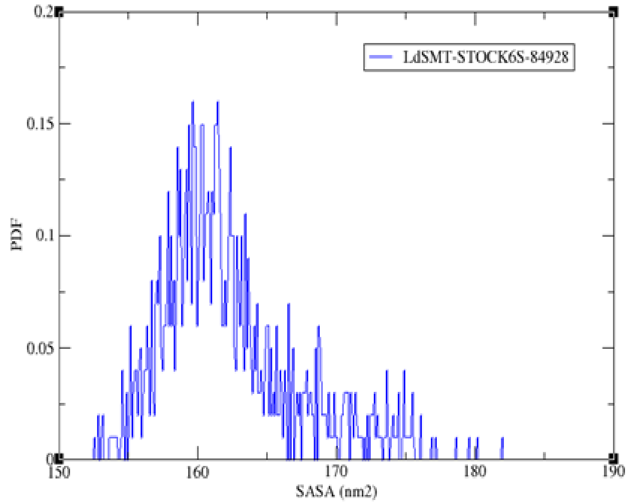

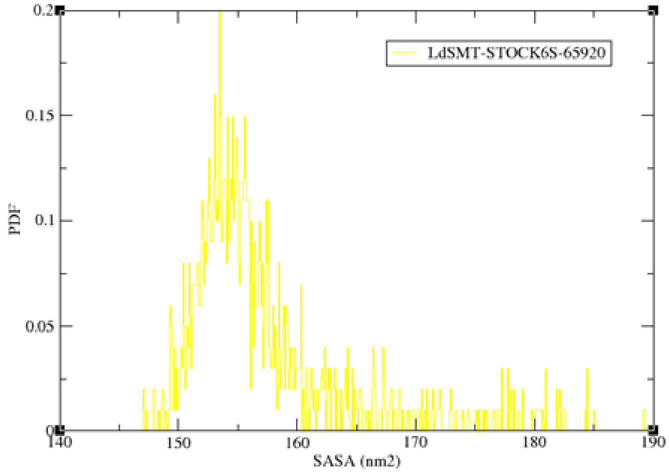

Figure S5. SASA analysis of 100 ns trajectory of the unbound *Ld*SMT, complexes of the three selected hits and 22,26-Azasterol. (a) SASA for unbound protein and all the complexes, (b) PDF of SASA for unbound *Ld*SMT, *Ld*SMT-22,26-Azasterol, *Ld*SMT-STOCK6S-06707, *Ld*SMT-STOCK6S-84928, and *Ld*SMT-STOCK6S-65920.

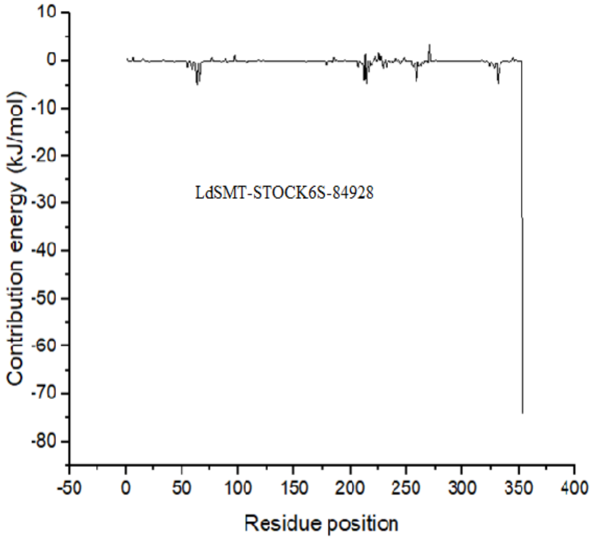

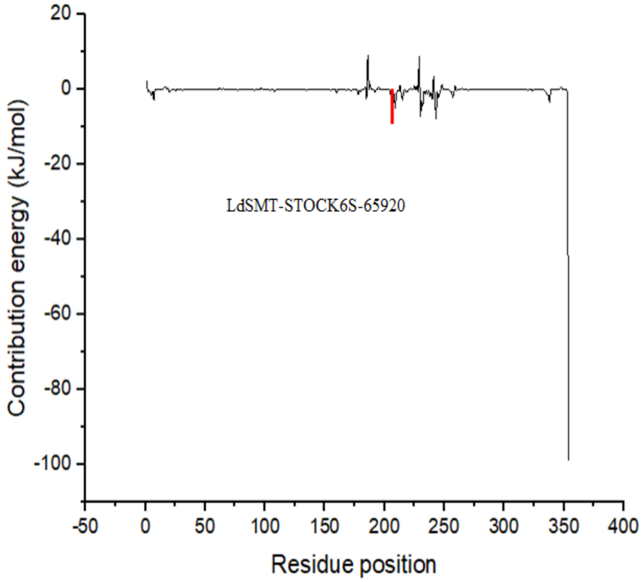

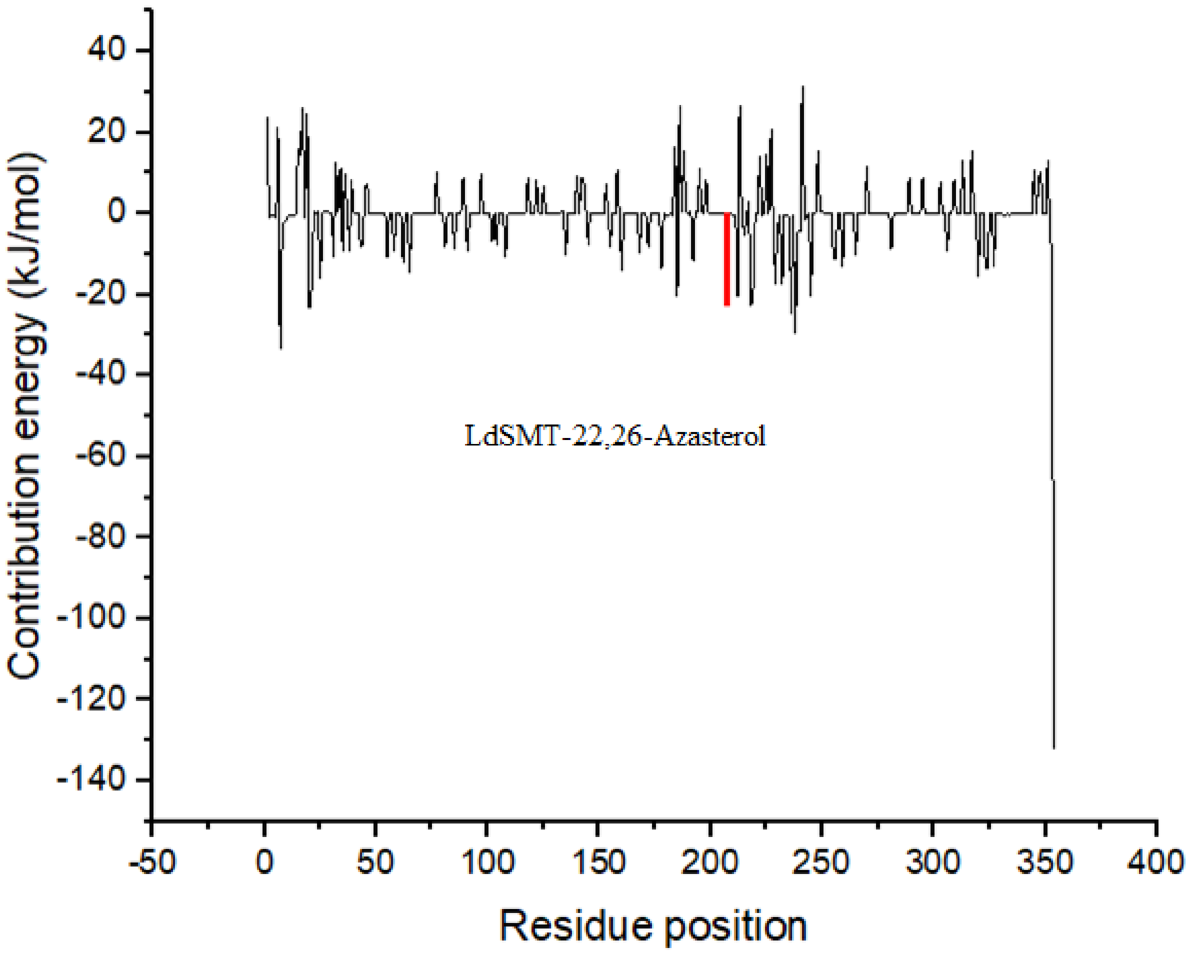

Figure S6. MM/PBSA computed per-residue energy decomposition for the *Ld*SMT-STOCK6S-84928, *Ld*SMT-STOCK6S-65920, and *Ld*SMT-22,26-Azasterol complexes.

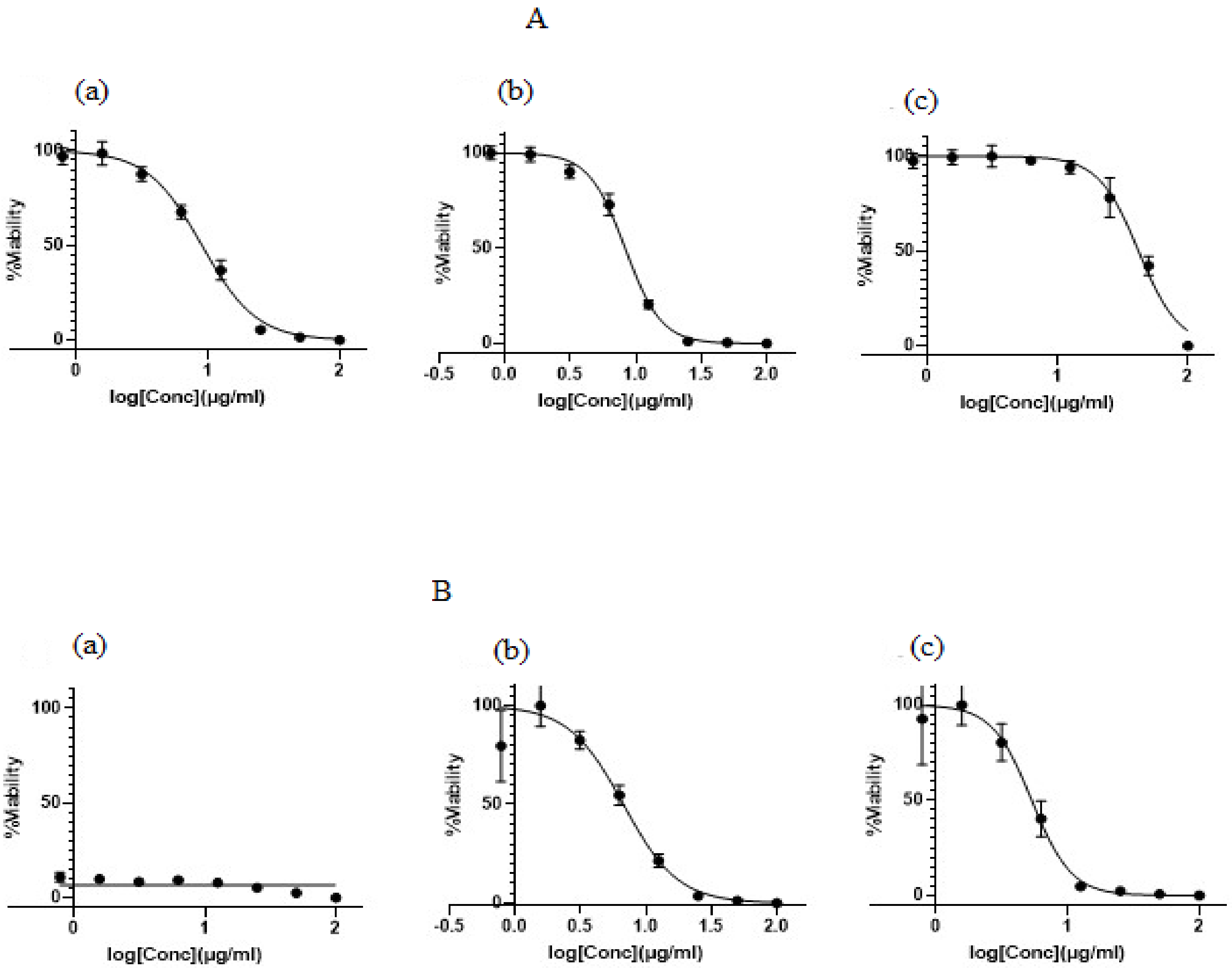

Figure S7. Graphical representation of the *in vitro* activity of hit compounds targeting (A) *Leishmania donovani,* and (B) *Trypanosoma brucei*. (a) STOCK6S-06707, (b) STOCK6S-65920 and (c) STOCK6S-84928. Data represent the mean of three independent biological replicates.
